# Statistical Modeling of the Abundance of Vectors of West African Rift Valley Fever in Barkédji, Senegal

**DOI:** 10.1371/journal.pone.0114047

**Published:** 2014-12-01

**Authors:** Cheikh Talla, Diawo Diallo, Ibrahima Dia, Yamar Ba, Jacques-André Ndione, Amadou Alpha Sall, Andy Morse, Aliou Diop, Mawlouth Diallo

**Affiliations:** 1 Unité d′Entomologie Médicale, Institut Pasteur de Dakar, Dakar, Sénégal; 2 Laboratoire d′Etudes et de Recherches en Statistiques et Développement, Université Gaston Berger, Saint-Louis, Sénégal; 3 Centre de Suivi Ecologique, Dakar, Sénégal; 4 Unité des arbovirus et virus de fièvres hémorragiques, Institut Pasteur de Dakar, Dakar, Sénégal; 5 School of Environmental Sciences, University of Liverpool, Liverpool, United Kingdom; University of Texas Medical Branch, United States of America

## Abstract

Rift Valley fever is an emerging mosquito-borne disease that represents a threat to human and animal health. The exophilic and exophagic behavior of the two main vector in West Africa (*Aedes vexans* and *Culex poicilipes*), adverse events post-vaccination, and lack of treatment, render ineffective the disease control. Therefore it is essential to develop an information system that facilitates decision-making and the implementation of adaptation strategies. In East Africa, RVF outbreaks are linked with abnormally high rainfall, and can be predicted up to 5 months in advance by modeling approaches using climatic and environmental parameters. However, the application of these models in West Africa remains unsatisfactory due to a lack of data for animal and human cases and differences in the dynamics of the disease emergence and the vector species involved in transmission. Models have been proposed for West Africa but they were restricted to rainfall impact analysis without a spatial dimension. In this study, we developed a mixed Bayesian statistical model to evaluate the effects of climatic and ecological determinants on the spatiotemporal dynamics of the two main vectors. Adult mosquito abundance data were generated from July to December every fortnight in 2005–2006 at 79 sites, including temporary ponds, bare soils, shrubby savannah, wooded savannah, steppes, and villages in the Barkédji area. The results demonstrate the importance of environmental factors and weather conditions for predicting mosquito abundance. The rainfall and minimum temperature were positively correlated with the abundance of *Cx. poicilipes*, whereas the maximum temperature had negative effects. The rainfall was negatively correlated with the abundance of *Ae. vexans*. After combining land cover classes, weather conditions, and vector abundance, our model was used to predict the areas and periods with the highest risks of vector pressure. This information could support decision-making to improve RVF surveillance activities and to implement better intervention strategies.

## Introduction

Rift Valley Fever (RVF) is an emerging arboviral disease that affects humans and domestic and wild ruminants. Rift Valley Fever mainly affects sheep, goats, and cattle by causing abortions in pregnant females and high mortality in neonates [1], [2] thereby resulting in substantial socio-economic losses in affected areas. Humans can be infected via contact with blood, organs, tissues, fetuses, and the excretions of infected animals, or by infected mosquito bites. Human infections can lead to severe diseases, which are associated with a high mortality rate [3], [4]. Rift Valley Fever virus (RVFV) is transmitted by a number of species of mosquito, primarily from the genera *Aedes* and *Culex* [5], [6], [7], and in the Barkédji area of Senegal, has been isolated from several mosquito species but most commonly from the abundant *Cx. poicilipes* and *Ae. vexans*.

These vectors breed in temporary ponds that are flooded after the first rains [7], [8]. *Aedes vexans* spend the unfavorable season as resistant desiccated eggs that hatch synchronously as soon as the ponds are flooded. Larval development is complete in less than 10 days [9] and adult survival is estimated to be up to 3 months [10]. Therefore, its population reach their peak of abundance at the beginning of the rainy season. *Culex poicilipes* spend the dry season as nulliparous mated females that lay eggs at the beginning of the rainy season. The population grow gradually to reach its peak of abundance later. Both vectors have their highest parity rates between September and October in Barkédji [11].

The maximum flight distances estimated for *Ae. vexans* and *Cx. poicilipes* from their larval sites were 620 and 550 m respectively [12]. These vectors were collected in all land cover classes investigated in the Barkédji area but they preferred barren and temporary ponds and were rare within villages indicating their exophilic and exophagic pattern [11]. The two vectors are considered to be opportunistic feeders with a zoophilic tendency. Their vertebrates host include wild animal species, livestock and equine living or divagating around the ponds. Reports suggest less than 1% of blood meals from *Ae. vexans* may be taken on human [13].

The transmission cycle includes an enzootic cycle that occurs around temporary ponds, where the virus maintained in *Aedes* eggs resistant to desiccation over several years. An amplification cycle occurs when the weather conditions are suitable for infected eggs hatching and proliferation of infected adult mosquitoes able to transmit the virus to livestock. In East Africa the virus emergence and the amplification cycle are known to be associated to unusual heavy rains [14]. In West Africa, the virus emergence is hypothesized as the consequence of infected eggs hatching from temporary ponds or virus introduction trough livestock mobility. The switch of the enzootic cycle to an epizootic/epidemic results from the combination of several factors including mosquito proliferation and dispersal [12], herd concentration and their contact with human population [5].

In humans, the only effective vaccine available is currently limited in terms of production, and it is restricted to at-risk personnel since it requires multiple inoculation to achieve protective immunity [15]. Several veterinary vaccine candidates have been proposed or are under investigation (MP12, Clone 13, Smithburn neurotropic strain, R566) [16], [17], [18], [19]. Some of these vaccines are not favored because of their abortive/teratogenic properties [20], [21], [22]. Their adverse events concern re-assortment potential as well as environmental safety, including the potential to be transmitted by vectors. Furthermore, the practical implementation of vaccination is challenging because of poor outbreak forecasting.

Similarly, larval control is difficult to envisage because of the nature of the breeding sites, including temporary ponds, which represent the main water sources for people, livestock, and other wildlife in the Barkédji area. The methods used by people for protection against adult mosquitoes (Long-Lasting Insecticide-treated Nets, aerosols, and insecticide coils) are also ineffective because of the exophagic and zoophilic behaviors of these RVF vectors [13]. Mosquito control can be achieved by several methods including thermal fogging, ultra-low volume ULV [23]. However, due to the extent of surface to be treated, potential environmental impact and the equipment required (aircraft, helicopter…), these methods are still inaccessible to low income countries. The treatment of animals with insecticide has shown promising results in different geographical areas, including Barkedji [24]. The acceptance by pastoralists of this control method, nevertheless, remains an obstacle for implementation and dissemination.

These considerations have motivated the use of modeling approaches to predict and prevent the risk of RVF emergence, as well as for developing information systems to support decision-making processes and the implementation of adaptation strategies. The knowledge of areas of high and low vector abundance, and the potential risk of infection, would allow the implementation and adaptation of targeted vector control strategies and also identify appropriate places for pastoralists to settle so that contact between vectors and hosts is minimized. It would also provide information to the authorities about which areas to treat, based upon small-scale insecticide use (thermal fogging or ultra-low volume (ULV) spraying, cattle treatment…).

The identification of these areas at risk could help to better plan and minimize risks associated with vaccination. Only herds located in at risk areas are vaccinated.

Variability in the weather and climate often influence the transmission of many infectious diseases, particularly those spread by arthropod vectors such as malaria and dengue [25]. Some vector-borne diseases exhibit seasonal patterns with inter-annual and intra-annual variability, which are explained partly by climate and environmental factors [26]. Therefore, the use of climate information in early warning systems for diseases could provide public health decision-makers with advance notice of the possible occurrence of a disease, thereby allowing the implementation of timely preventative measures such as education of persons at risk (herders, butcher, veterinary, and health workers), animal movement restrictions, targeted vaccination and/or ULV sprays. This type of early warning system requires statistical and/or biological models that incorporate the effects of climate variables on disease transmission.

This modeling approach was developed in East Africa based on the identification of climatic and environmental variables (Pacific and Indian Ocean sea surface temperature anomalies (SSTs), satellite normalized difference vegetation index anomaly) that control the occurrence of RVF. In Africa, an early warning system has been developed that can predict RVF outbreaks up to 5 months in advance using parameters such as the normalized difference vegetation index (NDVI) and sea surface temperature anomalies [27]. A retrospective study using time series analysis showed that this system was capable of accurately predicting three RVF outbreaks in Kenya between 1982 and 1998. However, it failed to predict the RVF outbreaks in Senegal during 1993, Burkina Faso in 1983, and Central Africa in 1985 [23], [28], [29], [30]. It may be due to a possible lack of sensitivity of the system like the threshold of the NDVI or a lack of data from animal cases for model validation. These findings suggested that this model was not suitable for use in West Africa, possibly due to the different dynamics and mechanisms of emergence in East and West Africa [31], lack of documented data for animal/human cases and vector species involved in the transmission (*Aedes mcintoshi* and *Culex pipiens* in East Africa versus *Aedes vexans* and *Culex poicilipes* in West Africa). While, *Aedes vexans* and *Aedes mcintoshi* are both floodwater zoophilic mosquitoes, *Culex pipiens* is more associated to human environment than *Culex poicilipes*. Rainfall is always an important climatic parameter that affects RVFV emergence in East Africa [27], but it does not always have the same effect in West Africa, i.e., years with rain deficits as well as excess are associated with RVFV emergence in West Africa. Instead, the parameter that appeared to have an important role was rather rains marked by long dry spells [12], [32]. Therefore, several models have been developed for the West African context at a local-scale [31], [33], [34], [35], [36] and a country level [37], but the analysis is restricted to the impact of rainfall in most of these models, which lack the spatial dimension. Furthermore, most of these models have been developed specifically for *Ae. vexans*, which is one of the main vectors. The only model developed for *Cx. poicilipes* that considers rainfall as a climatic parameter used entomological data from Barkedji but some biological parameters not available (sex ratio, number of eggs laid/female/day, daily larval survival rate, date of diapause, etc.) were estimated based on data relative to *Culex pipiens pipiens* in America exhibiting different dynamics [31]. In addition, this model did not integrate ecological and climate data such as the temperature and relative humidity, which affect the survival and development of mosquitoes. Therefore, the development of a model that integrates several ecological and climatic parameters may facilitate the establishment of an effective monitoring system for the Barkédji area. A system for monitoring and predicting the seasons and areas with high and low levels of vector abundance could support decision-makers in the implementation of effective control strategies.

In this study, therefore, we developed a mixed Bayesian statistical model to evaluate the effects of climatic and ecological determinants on the spatiotemporal dynamics of *Ae. vexans* and *Cx. poicilipes* at a local-scale. Based on the observed abundances and spatial distributions, this model was used to predict their geographic distributions using climatic and environmental data to identify potential risk areas.

## Materials and Methods

### Entomological data

**Figure 1:**
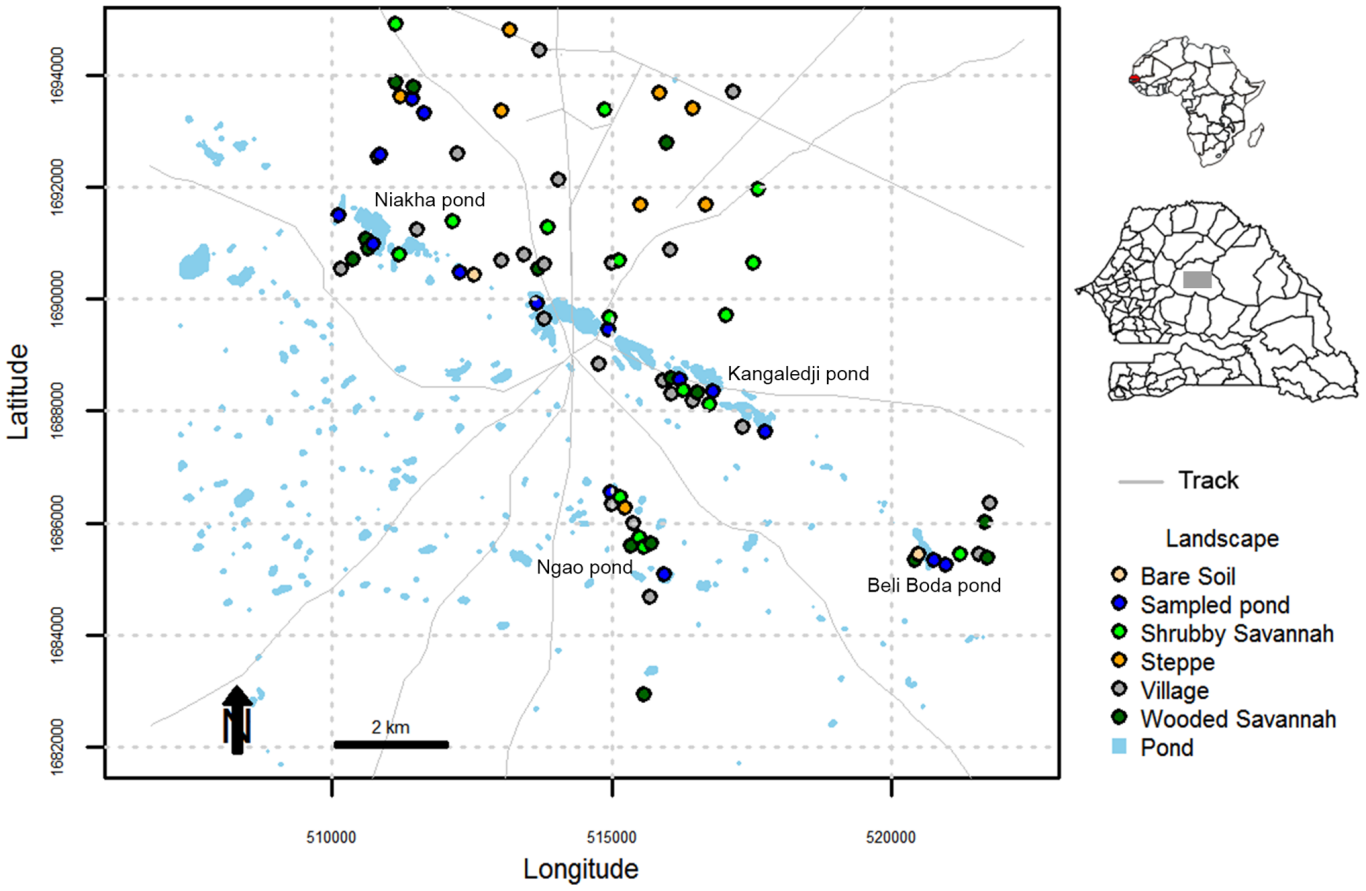
Study area.

The data used were obtained during a study conducted over a 2-year period (2005–2006) within 13 km around the village of Barkédji (14°47′–14°53°W, 15°13′–15°20′N) during the rainy season (Figure 1). This area belongs to the Sahelian zone and is characterized by a hot dry season (November to May) and a short rainy season (June to October), with an average annual rainfall of 300–500 mm. During the rainy season, a large number of small, temporary ponds develop to form a network. Different types of ponds are present in the Barkédji area, which have different levels of aquatic vegetation cover. For example, the ponds named Kangaledji and Niakha are heavily vegetated in (79% and 60% vegetation coverage, respectively) [38]. The large pools such as Niakha, Kangaledji, and Ngao are flooded for up to 3 months after the rainy season, and these temporary ponds are major water sources for people and livestock. They are also natural habitats for many species of birds, reptiles, and rodents, as well as providing favorable locations for the development (oviposition and resting) of mosquitoes that are potential vectors of RVFV. Barkédji is a stopover area for transhumant herds so the number and the spatial distribution of livestock in the area depend on the arrival or the departure of nomadic herds.

Mosquitoes were sampled from 79 sites that included the six major land cover classes identified in the area. High-resolution remote-sensing satellite data (SPOT 5, 10 m) were used to identify the different ecological land cover classes. The descriptions of the vegetation classes were based on a combination of the FAO (1997) [39] and Anon (1956) [40] systems, where the following environmental classes were selected: ponds, wooded savannahs, shrubby savannahs, bare soils, steppes, and villages. The land cover class was defined according to these six ecological classes and it was treated as a categorical variable or factor during modeling.

Mosquitoes were collected using Centers for Diseases Control light traps with CO2 dry ice one night from each site every 2 weeks between July and December 2005 and 2006, yielding a total of 22 sampling dates. Traps were set in each site at about 50 cm above ground level at the edge of temporary pond, near a house in villages and at the center for the other landscape classes. They were killed and identified morphologically using identification keys [41], [42]. Each sampling site was geo-referenced using a hand-held GPS receiver and the Euclidean distance (m) to the nearest pond was estimated using the SPOT 5 satellite image of the study area.

### Climate and environmental data

The climatic and environmental parameters considered in the present study were: rainfall, relative humidity, maximum temperature, minimum temperature, and NDVI. The minimum and maximum temperature have been selected because there is evidence that in nature, mosquitoes do not simply experience mean temperature, but are subjected to huge temperature fluctuations throughout the day. Recent studies showed that maximum and minimum temperature could impact differently the abundance of several mosquito species like *Aedes vexans, Culex pipens* and *Culex restuans* in America [43], [44].

During the study period, the rainfall, relative humidity, and maximum and minimum daily temperatures were collected by eleven automatic weather stations (1 BWS 200 Campbell Scientific and 10 HOBO weather stations) installed inside villages (for security reasons) and distributed evenly in the Barkédji area. For analysis data, each sampling site was associated to the nearest weather station. The spatial NDVI data were derived from the MODerate-resolution Imaging Spectroradiometer (MODIS|MOD13) on NASA's Terra satellite (National Aeronautics and Space Administration, NASA), where the NDVI was based on 16-day averages at a spatial resolution of 250 m. For each sampling site and each of 22 sampling periods, the corresponding NDVI values were downloaded from NASA website [45].

The correlation between the climatic and environmental explanatory variables was checked using Kendall's rank correlation [46], [47]. We also estimated the variance inflation factor (VIF) using a generalized linear model under a Gaussian distribution to detect the level of correlation among variables (Table S1). Collinearity generally should be considered in analyses when VIF is >10 [48].

### Statistical analysis

The associations between the abundance of each mosquito species and the climatic and environmental factors were analyzed using generalized linear mixed models (GLMMs).

The GLMM is an extension of the classical generalized linear model but it considers correlated data structures, including spatially unstructured and spatiotemporal structured random effects, in the linear predictor. We considered all of the explanatory variables in the generalized linear Poisson model. The model parameters were estimated within a Bayesian framework using the Markov Chain Monte Carlo (MCMC) method, where they were considered to be statistically significant if their 95% credible interval did not contain zero. The Bayesian approach accounts for parameter uncertainty by assigning prior distributions to the parameters [49]. An advantage of this approach is that the associated MCMC sampling yields samples from full posterior predictive distributions, which automatically incorporate all the components of variance at the different levels of the model. Therefore, a full assessment of the prediction uncertainty can be obtained more easily with the Bayesian MCMC estimation than the more traditional maximum likelihood approach. Given the known seasonal pattern in mosquito abundance, we introduced seasonality as a sinusoidal curve, which had the same peak of abundance as the mosquito species. The inclusion of random effects in the model framework allowed us to account for unknown or unobserved confounding factors in the disease system by introducing an extra source of variability into the model [50]. Unstructured random effects could help to account for overdispersion in the distribution of the mosquito counts obtained. However, this did not explain the spatial dependence of the numbers collected at different points. This dependence was considered in the model by adding spatially structured random effects. The typical choice for spatially structured random effects is an intrinsic Gaussian conditional autoregressive (CAR) model [51]:
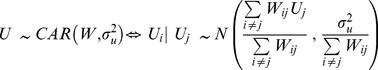
(1)where 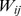 are adjacency weights for sites that take binary values, i.e., 1 if site i and site j are considered neighbors but 0 otherwise. We used point data and the nearest neighbors was determined using Euclidian distance. Variables 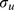 and 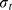 control the intensity of the local spatial and temporal dependence respectively. An autoregressive temporal 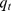 effect is included in the model, where t represents the fortnightly catch for each month and 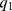 is set equal to 0 (second fortnight in July) to avoid identifiability problems in the model. The spatiotemporal GLMM formulated to assess the importance of climatic and non-climatic variables is described as follows:

*Ae. vexans *model

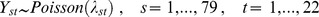


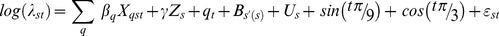
(2)

*Cx. poicilipes* model

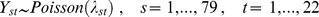


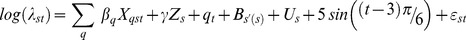
(3)

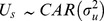
(4)

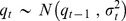
(5)


For both models, 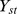 represents the abundance of each species assumed to follow a Poisson distribution at site 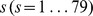; and fortnights 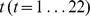, 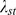 is the corresponding mean number of individuals collected for each species. The parameter 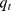 defined in equation (5) is a first order auto regressive temporal effect. The parameter 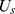 specified in equation (4) represents an intrinsic Gaussian CAR distribution defined above in equation (1). The parameter 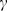 is the regression coefficient for 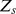 (distance between a collection point and the nearest pond). The variable 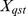 represent the climatic and environmental variables considered, i.e., rainfall 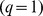, which represents the cumulative rainfall (total precipitation) 15–20 days prior to trapping, where this lagged variable was selected to take into account the cumulative effect of rain on mosquito productivity and abundance; maximum temperature 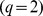, minimum temperature 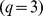, relative humidity 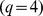, and NDVI 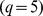. The rainfall lag time was suggested by the field data recorded every fortnight. Analyzing the population dynamics of vectors (Figure S1) we observed a lag time of at least 15 days between the peaks of rainfall and the peak of vectors abundance [11]. In addition a previous study revealed the correlation between rainfall and *Ae. vexans* abundance 10 days after rains at the beginning of the rainy season and 20 days at the end [52].

To standardize the approach, the same periodicity was used to analyze the climate and environmental variables. In addition, this lag time and suitable weather forecasting may provide public health decision-makers with sufficient time to prepare for and respond to an outbreak.

The variable 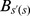 represents the six land cover classes described above, 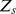 is the distance between the sampling point and the nearest pond, and 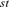 is the interaction between the site and the collection period. All of the computations were based on posterior samples derived using MCMC methods. Two chains were run with 35,000 iterations each and the first 30,000 iterations of each chain were discarded to eliminate the possible dependence on the initial values, where the sampling interval (thinning value) was 10, thereby yielding a random sample of length  = 1,000 for each parameter in the model. We used the “Coda” library from the R statistical package [53] to perform the convergence test with the Gelman and Rubin statistic [54], where the statistic was checked if it was <1.1 with an effective sample size >150 for all parameters. The complete model specification is described in detail in the supporting file (Model S1).

### Model selection

The goodness of fit was assessed for all the models tested using the deviance information criterion (DIC) [55], [56], which is a widely used Bayesian selection criterion for selecting models based on the requirement to reach a compromise between the goodness of fit and model complexity. The DIC is based on the sum of the posterior mean deviance and the number of effective parameters used in the model [55]. Smaller values of DIC indicate a model with a better fit, thereby indicating good performance in predicting unobserved quantities [57]. The models with the lowest DIC values were considered as the best models. They provide the best explanation of the data and were selected to predict the abundance of each mosquito species.

In addition, we computed Pearson's correlation coefficient to evaluate the agreement between the observed and expected mosquito abundance values, as well as the root mean squared error (RMSE) to measure the precision, where the predicted numbers were calculated based on estimates of the posterior means 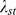. The models were generated using R software [58] and the R2WinBUGS package [59] in conjunction with the free statistics software WinBUGS 1.4.3 [60] to perform computations based on posterior samples derived via MCMC methods for parameter estimation in the GLMM model.

We generated raster maps of the predictions, which showed the mosquito abundance for each pixel in the study area, by applying the inverse distance-weighted interpolation function [61] using “gstat” library [62] in R statistic software. This function measured the mosquito abundance for each point in the study area and represented it as a raster file.

## Results

### Mosquito collections

In total, 29,183 female mosquitoes from 39 species were collected during 1,738 trap-nights. *Culex poicilipes* was the most abundant species (28%) followed by *Ae. vexans* (21%). Rift Valley Fever vectors (*Ae. vexans, Ae. dalzieli, Ae. fowleri, Ae. ochraceus, Cx. poicilipes, Mansonia africana, Mansonia uniformis*) represented about 68% of the total mosquitoes collected. *Aedes vexans* and *Culex poicilipes*, the two main vectors, represented 73% of the population of RVF vectors. Both species exhibited seasonal variability in abundance (Figure S1). *Aedes vexans* had two abundance peaks in the second half of July at the beginning of the rainy season (in 2005 and 2006) and during the first fortnight of October in 2005, and the second fortnight of September in 2006. For *Cx. poicilipes*, the peaks occurred during the first fortnight of September and the second fortnight of October in 2005, and during the first fortnight of October in 2006. *Culex poicilipes* was most abundant around ponds and wooded savannahs, whereas the distribution of *Ae*. *vexans* appeared to be more uniform among different land cover classes (Figure S2A–B).

The annual rainfall were 458.9 mm in 2005 and 410.5 mm in 2006, where July was the wettest month with a total of 189 mm in 2005, but August was the wettest month with 157.4 mm in 2006. Rainfall stopped during the first half of October in both years. The daily temperature was highest in the second half of October at 45.4°C during 2005 and lowest during the first half of December at 12.6°C in 2006. The relative humidity was highest in the second and the first half of September respectively in 2005 and 2006 (81.4% in both years). The NDVI was highest during the first half of September in both years (0.45 in 2005 and 0.39 in 2006) (Figure S3).

### Model results

The results of VIFs and Kendall's rank correlation coefficient for all climatic and environmental variables indicated no significant collinearity (Table S1). After convergence diagnosis, the final models were constructed and the parameter estimates for the GLMM models for each species are summarized in Table 1. Spatially structured random effects were not present in the parsimonious model for *Ae. vexans*. The posterior distributions for each climatic and environmental parameter that differed from zero, and the signs of the associations with vector abundance in the models are shown in Figure S4A–E and Figure S5A–B. Thus, the abundance of *Cx. poicilipes* was significantly and positively associated with the average minimum temperature and the cumulative rainfall recorded 15–20 days prior to sampling. However, the average maximum temperature and NDVI index were significantly and negatively associated with the abundance of this vector. The distance from the nearest pond as well as land cover classes in equation (3) and the CAR distribution in equation (2) were not statistically significant and they had no influence on the best model for each species. The average relative humidity had an effect on the best model but it was not statistically significant.

**Table 1:** Posterior mean, convergence diagnostic 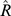 for covariates and hyperparameters associated with temporal and spatial random effects.

			mean	sd	2.5%	97.5%	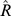
*Cx. poicilipes*	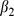	Min temperature	0.7746	0.0502	0.6773	0.8737	1.00
	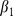	Max temperature	−0.2885	0.0193	−0.3282	−0.2527	1.00
	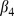	Rainfall	0.2246	0.0288	0.1710	0.2820	1.00
	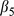	NDVI	−0.1999	0.0296	−0.2582	−0.1438	1.01
	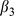	Relative humidity	−0.0651	0.0523	−0.1685	0.0381	1.00
	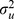	Spatial structured parameter	18.13	3.358	12.79	25.99	1.00
	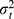	Temporal structure parameter	1.243	0.3445	0.76	2.08	1.00
*Ae. vexans*	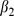	Delta	−0.0327	0.0073	−0.0471	−0.0182	1.00
	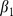	Rainfall	−0.0045	0.0005	−0.0054	−0.0035	1.00
	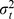	Temporal structure parameter	1.15	0.355	0.682	1.977	1.00

NB: DIC value for *Culex poicilipes*  = 15324.4; DIC value for *Aedes vexans*  = 5901.82. Mean: posterior mean, sd: posterior standard deviation, 2.5% and 97.5%: quantiles of the distribution provide the credible interval, Delta  =  Max temperature-Min temperature.

The abundance of *Ae. vexans* was significantly and negatively associated with cumulative rainfall during 15–20 days before the collection period. The difference between the average maximum and minimum temperatures was negatively correlated with the abundance of this vector, whereas the effects of the NDVI index and the average relative humidity were not statistically significant.

The GLMM models for the two species had high predictive power (RMSE  = 4.1 and RMSE  = 20 for *Ae. vexans* and *Cx. poicilipes*, respectively) in capturing the seasonality and spatial distribution of each species. The observed and expected mosquito numbers had similar patterns with a high correlation (Pearson's correlation coefficient: r = 0.99, *P*<0.0001; r = 0.58, *P*<0.0001; for *Ae. vexans* and *Cx. poicilipes*, respectively).

Figure S6A–B shows the relationship between the observed data and abundances predicted by the GLMM models, which suggests that most of the variability in mosquito abundance was simulated well by the models, although the numbers of individuals collected tended to be underestimated during the period of peak abundance.

**Figure 2:**
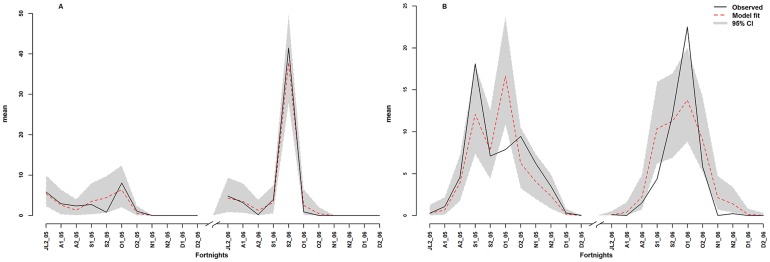
Temporal pattern of observed and predicted abundance. Observed and predicted for *Cx. poicilipes* (A) and for *Ae. vexans* (B).

**Figure 3:**
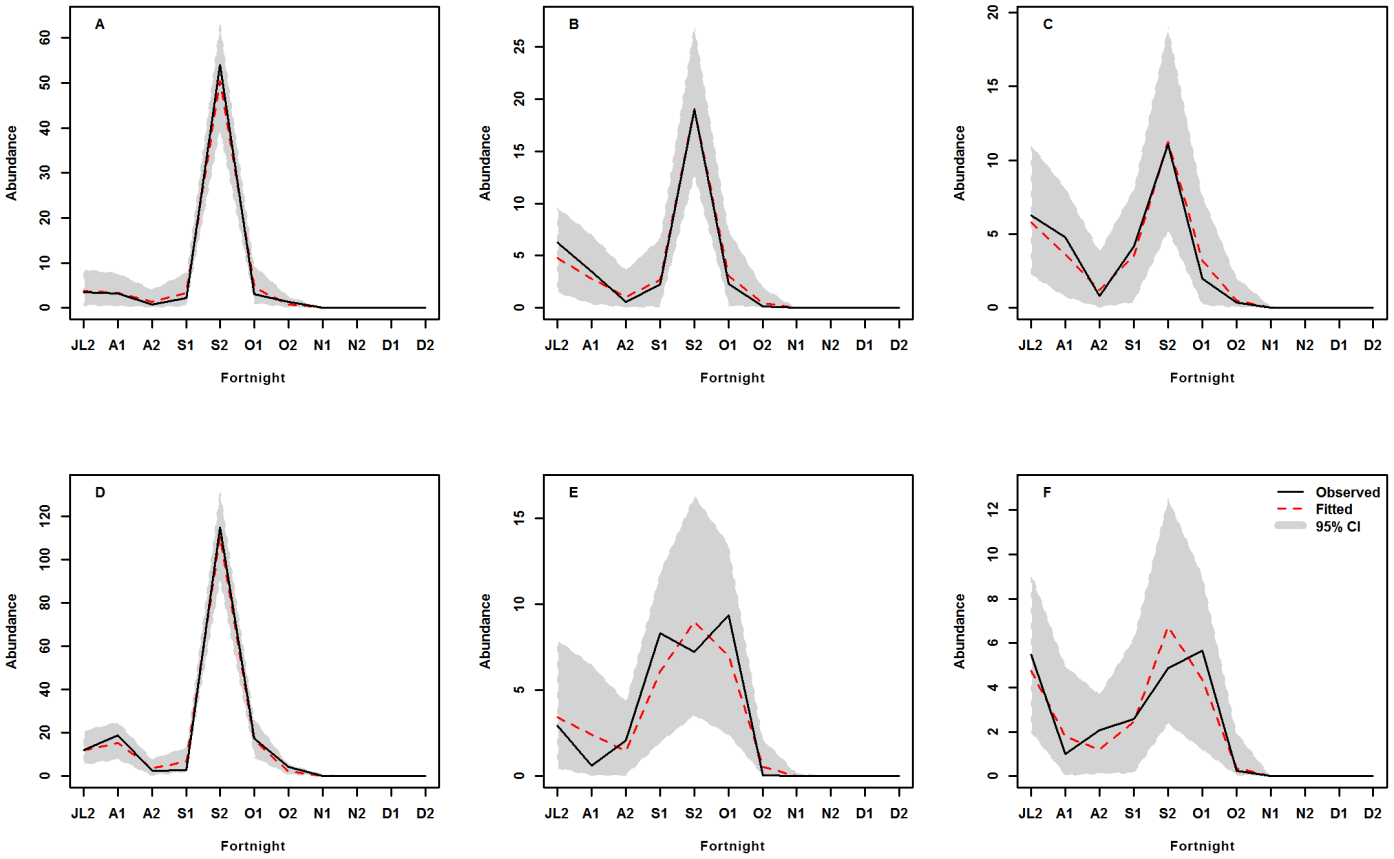
Temporal pattern of observed and predicted abundance by land cover class. Observed and predicted for *Ae. vexans*, A: pond, B: Wooded savannah, C: shrubby savannah, D: Bare soil, E: Steppe, F: Village.

To assess the predictive capacity of the model, the posterior predictive distributions (posterior predictive mean and 95% prediction intervals) of the abundances of the two vectors were simulated based on the parameters estimated using the two best models (considering unknown random effects) in terms of the number of individuals collected, all land cover classes combined (Figure 2A–B), and each land cover class for *Ae. vexans*. The models captured correctly the intra-seasonal variability in the abundance of two important vectors, which has implications for disease risk in areas where the virus is circulating, even if in some cases the model underestimated or overestimated the number of individuals compared with the field data. The predictions of the best model for *Ae. vexans* captured the variability in the observed data correctly during the period of peak abundance in the different land cover classes (Figure 3A–F).

### Vector risk map

**Figure 4:**
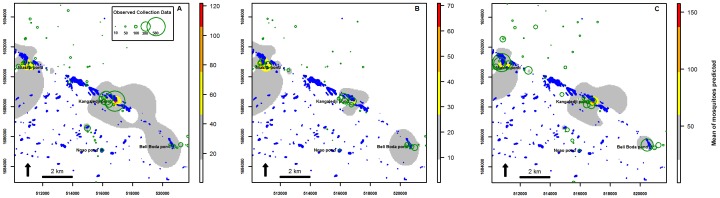
Prediction maps. Prediction for *Cx. poicilipes* during peak abundance in September 2005 (A), in October 2005 (B) and October 2006 (C). The observed collection data are represent in green circle.

**Figure 5:**
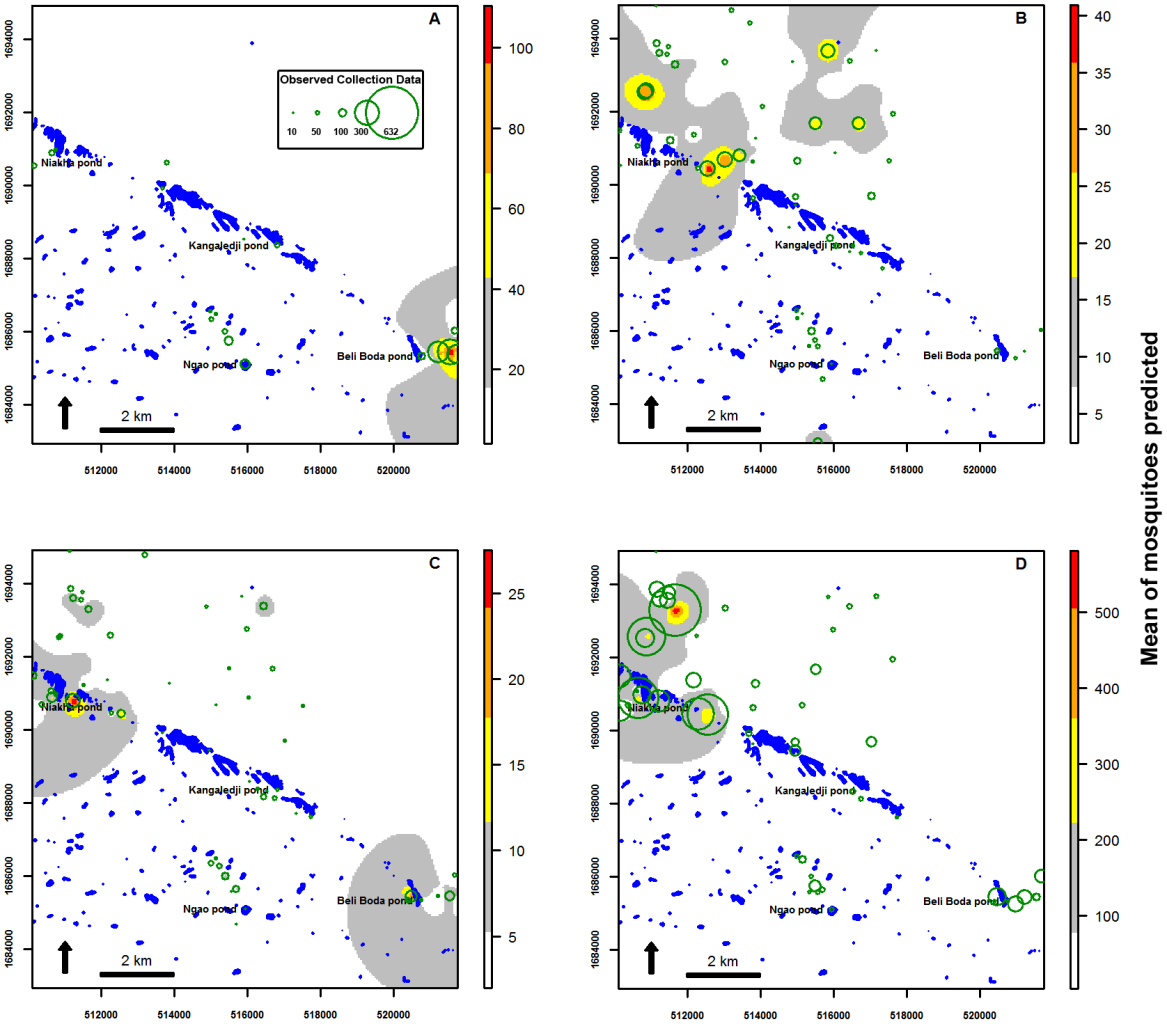
Prediction maps. Prediction for *Ae. vexans* during peak abundance in July (A) and October (B) in 2005 and in July (C) and September (D) in 2006. The observed collection data are represent in green circle.

The GLMM model for each vector was used to generate a prediction map for the period of peak abundance. The highest abundance of *Cx. poicilipes* was predicted to occur in close proximity to Kangaledji and Niakha ponds during the abundance peaks, i.e., in the first fortnight of September (Figure 4A) and second fortnight of October (Figure 4B) in 2005, and the first half of October in 2006 (Figure 4C). In 2005, the predicted distribution of *Ae. vexans* showed that this species was abundant near Beli Boda pond during the second fortnight of July (Figure 5A) and in the Northern and Eastern part of Niakha pond during the second abundance peak in October (Figure 5B). In 2006, the highest abundance of *Ae. vexans* was predicted near Niakha and Beli Boda ponds during the second fortnight of July (Figure 5C) and in close proximity to Niakha pond, in the second half of September (Figure 5D).

## Discussion

The impacts of spatially heterogeneous environmental and climatic factors on mosquito population dynamics are complex and poorly understood. In particular, for vector-borne diseases such as RVF, understanding the interactions between the vectors, hosts, and their environment may provide useful insights into the conditions that are suitable for pathogen transmission and amplification. Previous studies in the Barkédji area have shown that *Ae. vexans* and *Cx. poicilipes* are the two main vectors involved in the transmission of RVFV in terms of virus isolation, bio-ecology, and abundance [11], [63]. Switches in dominance between *Cx. poicilipes* and *Ae. vexans* in the Barkédji area were observed on a regular basis between 1990 and 1995 [8], but the factors involved in these changes and their impacts on the epidemiology of RVF remain unknown [11]. The analysis of a longitudinal dataset based on the abundance of these two main vectors, as well as the environmental and ecological conditions that are favorable for their development, may facilitate the prediction of their spatial occurrence and the identification of areas that could be suitable for the emergence and amplification of RVF. In this study, we determined the impacts of climatic and environmental variables on the abundance of these vectors. In contrast to a previous study where a significant impact was determined [11], the association between the abundance of RVF vectors and the distance to the nearest pond was not significant in the present study. This may have been due to the singular use of this parameter in previous study while in our models it was associated with several other ecological and climatic parameters. Rainfall was significantly associated with the abundance of *Ae. vexans*, as demonstrated by the significant negative correlation. This result contrasts with a previous study [11] using the same data that revealed a positive correlation between rainfall and the abundance of *Ae. vexans*. These studies used different approaches. In the study of Diallo et al. [11], the cumulative rainfall during the fortnight was considered and analyzed alone for each year. In our study, the cumulative rainfall 15–20 days before the collection period was used and associated with other parameters in the models. Such contradiction have been also exhibited by other studies using different approaches to analyze *Ae. vexans* abundance in Michigan [44].

These findings highlight the complexity of the relation between rainfall and *Ae. vexans* population dynamic. Several studies suggested that it is rather the first rain and those associated with breaks that positively impact the vector abundance [12], [52]. In addition a thorough observation of rainfall pattern in Diallo et al. [11] suggests that heavy rainfall recorded between August and September 2005 impact negatively the population of *Ae. vexans* after the peak recorded on July, while in 2006, *Ae. vexans* abundance peak was observed one month after the rainfall peak. This last observation is in contradiction with the species biology whose larvae survive and become pupae less than 10 days [9] suggesting the influence of other parameters.

Previous studies showed that between 1961 and 2003, RVF outbreaks did not coincide with years of heavy rainfall [31] in West Africa and the abundances of *Ae. vexans* and *Cx. poicilipes* were not correlated with the total rainfall [31]. Rainfall variability has been proposed as a key factor that affects mosquito populations and the abundance of *Ae. vexans* is considered to be dependent on the alternation of rainy and dry periods [12]. The difference between maximum and minimum temperature was negatively associated with the abundance of *Ae. vexans*, which suggested a temperature range favorable to the development of this vector.

All of the climatic and environmental variables included in the model were significantly associated with the abundance of *Cx. poicilipes*. The cumulative rainfall 15–20 days prior to trapping was positively associated with the number of *Cx. poicilipes* individuals collected. Indeed, although the level of precipitation decreased throughout the rainy season after a peak in July, the population of *Cx. poicilipes* increased gradually to reach its peak later at the end of the rainy season in strong association with floating vegetation [12], [64]. This was probably attributable to the biology of this species because few females of *Cx. poicilipes* spend the dry season (period when typically, all the ground pools are completely dry) as nulliparous mated females. These females become active at the beginning of the rainy season and rebuild the following *Culex poicilipes* population [11]. A previous study noted that there was a lag time of at least 1 month before observing the significant positive impact of rainfall on the abundance of *Cx. poicilipes* [11]. These observations indicate that rainfall has indirect effects and other factors may be associated with the abundance of this species given that the larval development cycle is <15 days [7]. Recently, it was suggested that the filling dynamics of temporary ponds (conditioned by the rhythm of rain) explain a large amount of the observed temporal variability in mosquito abundance [31], where the distribution of rainfall events is connected with the water level of the ponds [65]. Even if these elements are positively correlated with the abundance of *Cx. poicilipes*, however, they are not sufficient to explain the distribution dynamics because it is known that several ponds with the same rainfall pattern can have different production levels for this species. Therefore, other parameters with dynamics that may be linked to rainfall should be considered, such as the pond vegetation cover and the water quality, which are probable factors that affect the production of this species [12], [65].

We detected a negative association between the NDVI and the abundance of this vector. The NDVI was associated with a RVF outbreak in East Africa where anomalous high values were significantly correlated with RVF activity, and they predicted outbreaks 1–2 months before the detection of viral activity [27]. This is also supported by other studies, which show that the vegetation biomass surrounding ponds is a risk factor for RVF transmission regardless of the buffer size used in the calculation [66]. This result supports the assumption that the dense vegetation coverage on ponds is a favored mosquito resting site, which promotes their dispersion [67].

The maximum temperature was negatively associated with the number of *Cx. poicilipes* individuals collected. In contrast to the average minimum temperature, this was the most important parameter based on its strong positive correlation with the abundance of *Cx. poicilipes*. The correlation coefficient that reflected the association between the minimum temperature and numbers of mosquitoes collected was larger than that for the maximum temperature, which suggests that variations in the minimum temperature may have more effect than those in the maximum temperature. The antagonistic effect of the minimum and maximum temperatures has been reported in previous studies on malaria transmission, the parasite development, and the essential elements of mosquito bionomic. Indeed, in Ethiopia, Alemu et al. [68] showed that monthly total malaria cases were positively correlated with monthly minimum temperature and negatively with monthly maximum temperature. *Culex pipiens* abundance was positively associated with the preceding minimum temperature in the early season but negatively associated with maximum temperature in July and August, in a study conducted between 1989–2005 in Michigan [44]. Furthermore, cooler temperature acted to speed mosquito development, increased relative survival, and parasite growth rate, whereas warmer temperature caused an opposite effect [69], [70].

Thus, the abundance of *Cx. poicilipes* may be associated with low temperatures. This phenomenon appeared to be constrained by time, however, because our results also showed that although the decreases in the minimum temperature and maximum temperature were almost constant from the end of October, the population of *Cx. poicilipes* declined dramatically. The relative humidity may explain this decline in *Cx. poicilipes* populations during this period because it decreased significantly at the end of the rainy season. Indeed, the effects of all these parameters were delayed. The maximum and minimum temperature affected the vector abundance during the rainy season, but the relative humidity had the main effect at the end of the rainy season, irrespective of the temperature variations. Other studies in the Sahel area support this finding because they showed that high and moderate relative humidity levels did not affect the survival rates of *Anopheles gambiae* during the rainy season [71]. In the present study, however, the decline in the mosquito population at the end of the rainy season was explained by the relative humidity and not by the temperature, where the change in relative humidity was associated with a dramatic reduction in the number of mosquitoes during early November. When the relative humidity was 42%, the survival decreased to a critical value of 5%, where no individual could survive for longer than 24 h [71], thereby indicating that the relative humidity may determine the abundance of mosquitoes at the end of the rainy season. Indeed, low humidity reduces the survival of vectors because of dehydration and it may lead to an increase in the number of blood meals in an attempt to compensate for water loss [72].

Moreover, the abundance of *Cx. poicilipes* reached its peak during the rainy season when the relative humidity and vegetation index were high, and the gap between the maximum and minimum temperatures was great. The positive effect of the thermal range on the development of this vector during this period has also been reported for other insects, such as *Symmetrischema tangolias* in South America [73]. This observation is particularly important because recent studies have detected biases in many of the entomological and biological parameters that are estimated to characterize transmission, where the usual approach is based strictly on the mean monthly temperatures [74]. For malaria, it has been shown that the mean monthly temperatures overestimate or underestimate the extrinsic development of *Plasmodium* in warmer or cool conditions, respectively [74]. Furthermore, recent studies have demonstrated the influence of daily temperature variations on the biology of the vector and parasite. In particular, it has been shown that the important parameters governing malaria transmission (vector survival, duration of the gonotrophic cycle, development and survival of the aquatic stages, parasite development in the vector, and the extrinsic incubation period of the parasite) are sensitive to daily temperature fluctuations [69], [70]. The impact of daily temperature fluctuations on the vector's susceptibility to virus infection as well as vector survival have also been established for dengue [75].

*Aedes vexans* and *Culex poicilipes* were collected from all of the land cover classes considered in this study, but a previous study suggests that they are rare in domestic environments [12]. Both species preferred bare soils and temporary ponds to other land cover classes [11] because of their feeding preferences and the availability of vertebrate hosts around temporary ponds.

To assess this hypothesis, we used the best models for the two species to generate prediction maps for their peak abundance periods. The highest abundance of *Cx. poicilipes* was predicted to occur in close proximity to Niakha and Kangaledji ponds during the peak abundance periods of this vector. These two ponds are located in the Ferlo fossil valley where there is a high level of aquatic vegetation cover at the end of the rainy season. Previous studies in the Barkédji area have identified a total of 468 ponds on July in 2003 (the size of 80% of the individual ponds were less than 0.5 ha, whilst 2.3% were larger than 5 ha) [76] and 1354 ponds on August during the rainy season [38]. Ponds that are densely covered or shaded by vegetation are considered to be favorable larval habitats for *Cx. poicilipes* [67]. For *Ae. vexans*, high numbers of adult mosquitoes were predicted around Beli Boda pond during the second half of July at the beginning of the rainy season, as well as in the northern part of the study area and in the northern and eastern parts around Niakha pond. These finding indicate that the areas of interest for mosquito activity during peak abundance periods are the ponds in Niakha, Kangaledji, and Beli Boda, thereby highlighting the importance of implementing control programs in these areas. The map also showed that the northern part of the study area is one of the most important locations, which was predicted to have a high abundance of mosquito vectors.

In this study, we developed a model to integrate climatic and ecological parameters with effects on mosquito abundance, which have previously been considered separately. The model determined different spatially heterogeneous distributions for the two main vectors of RVF in the Barkédji area. Our predicted vector distribution maps highlight areas with high potential risks of RVF emergence, which can support surveillance and control programs. By combining land cover classes, weather conditions, and the activity levels of the vectors, our model predicted the areas and periods with the highest risk of vector pressure. This information could support decision-making by stakeholders, as well as helping end users to improve surveillance methods and to implement better intervention strategies. This is especially important for African countries where resources are scarce and appropriate guidance is essential for preventing mosquito bites or reducing vector abundance. This study may contribute to the development of an early monitoring system and it could be improved by considering human and animals populations exposed to RVFV.

## Supporting Information

Figure S1 **Number of mosquitoes collected every fortnight and cumulative rainfall.** Total mosquitoes collection every fortnight and cumulative rainfall 15–20 days prior trapping (Precipitations).
(TIFF)

Figure S2 **Spatial distribution of vectors abundance.** Spatial distribution (sum of mosquitoes collected): (A) *Cx. poicilipes* in 2005, (B) *Ae. vexans* in 2005, (C) *Cx. poicilipes* in 2006, (D) *Ae. vexans* in 2006.
(TIFF)

Figure S3 **Climate and environmental parameters used in the models.** Mean values for NDVI, Maximum/Minimum temperature and relative humidity. Cumulative rainfall 15–20 days prior to trapping.
(TIFF)

Figure S4
**Kernel density estimates for marginal posteriors distributions of parameters** 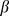 **associated with variables.** Posteriors distributions (posterior mean in parentheses): (A) Maximum temperature, (B) Minimum temperature, (C) Relative humidity, (D) Cumulative rainfall, (E) NDVI for *Cx. Poicilipes*.(TIFF)

Figure S5
**Kernel density estimates for marginal posteriors distributions of parameters** 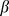 **associated with variables.** Posteriors distributions (posterior mean in parentheses): (A) thermal range (difference between maximum and minimum temperature), (B) Cumulative rainfall for *Ae. vexans*.(TIFF)

Figure S6 **Scatter plot and loess curve.** Show observed and predicted data (loess curve in solid line) using GLMM: (A) *Cx. poicilipes*, (B): *Ae. vexans*.
(TIF)

Table S1 **Kendall's rank correlation coefficients and the variance inflation factors (VIFs).** Variables correlated at less than 0.8 were maintained in the model. All the VIFs of climatic and environmental variables are well below 10 suggesting that collinearity is no longer a major issue.
(DOC)

Model S1 **WinBUGS codes for the Bayesian hierarchical models.**
(DOC)
