# Supplementary figures and images for: Statistical Modeling of the Abundance of Vectors of West African Rift Valley Fever in Barkédji, Senegal
Source: PLoS One. 2014 Dec 1;9(12):e114047. doi: 10.1371/journal.pone.0114047 (PMC4250055; doi:10.1371/journal.pone.0114047)

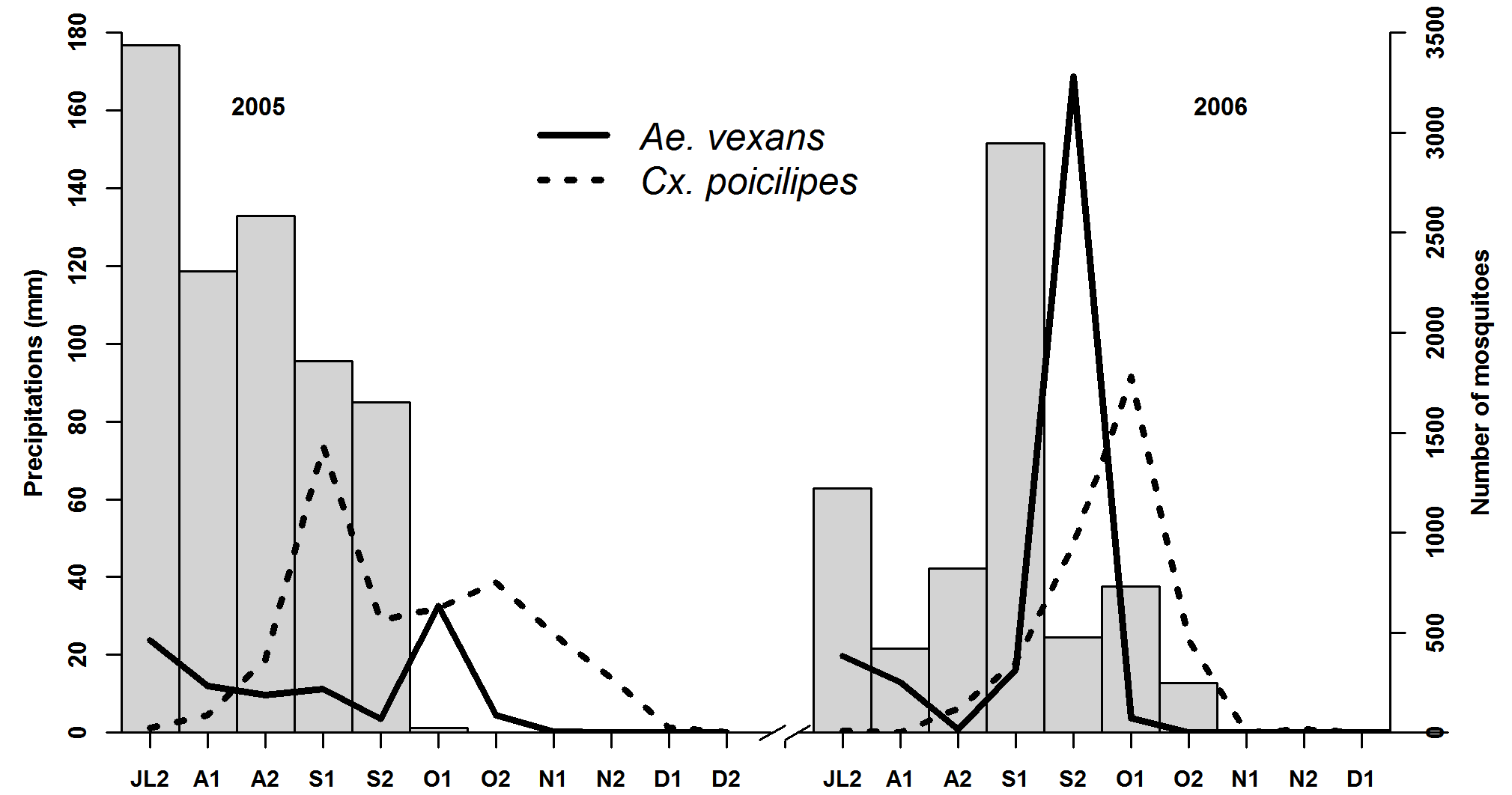

Supplement: Figure S1 — Number of mosquitoes collected every fortnight and cumulative rainfall. Total mosquitoes collection every fortnight and cumulative rainfall 15–20 days prior trapping (Precipitations). (TIFF) [file pone.0114047.s001.tiff]

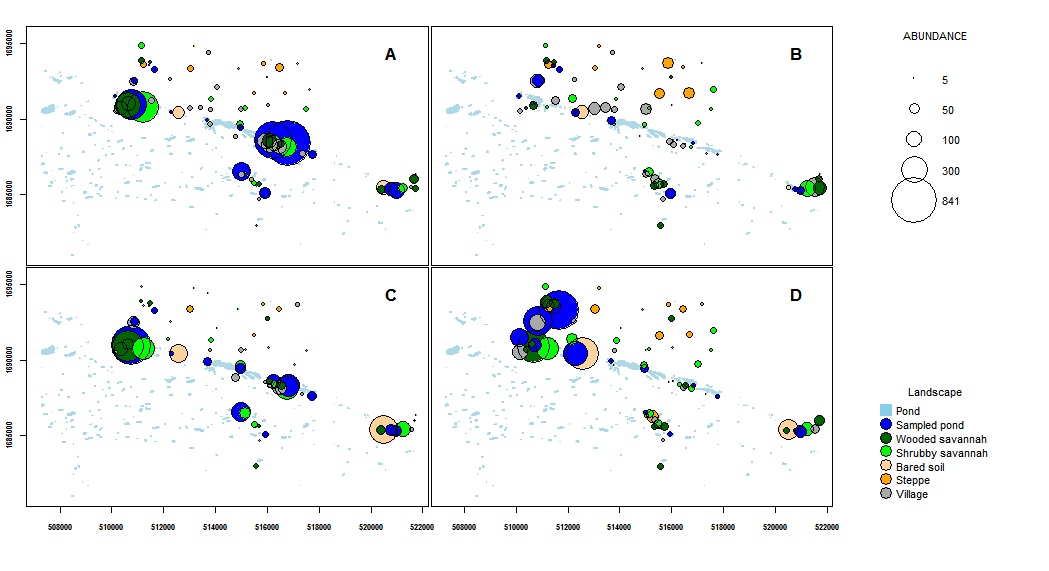

Supplement: Figure S2 — Spatial distribution of vectors abundance. Spatial distribution (sum of mosquitoes collected): (A) Cx. poicilipes in 2005, (B) Ae. vexans in 2005, (C) Cx. poicilipes in 2006, (D) Ae. vexans in 2006. (TIFF) [file pone.0114047.s002.tiff]

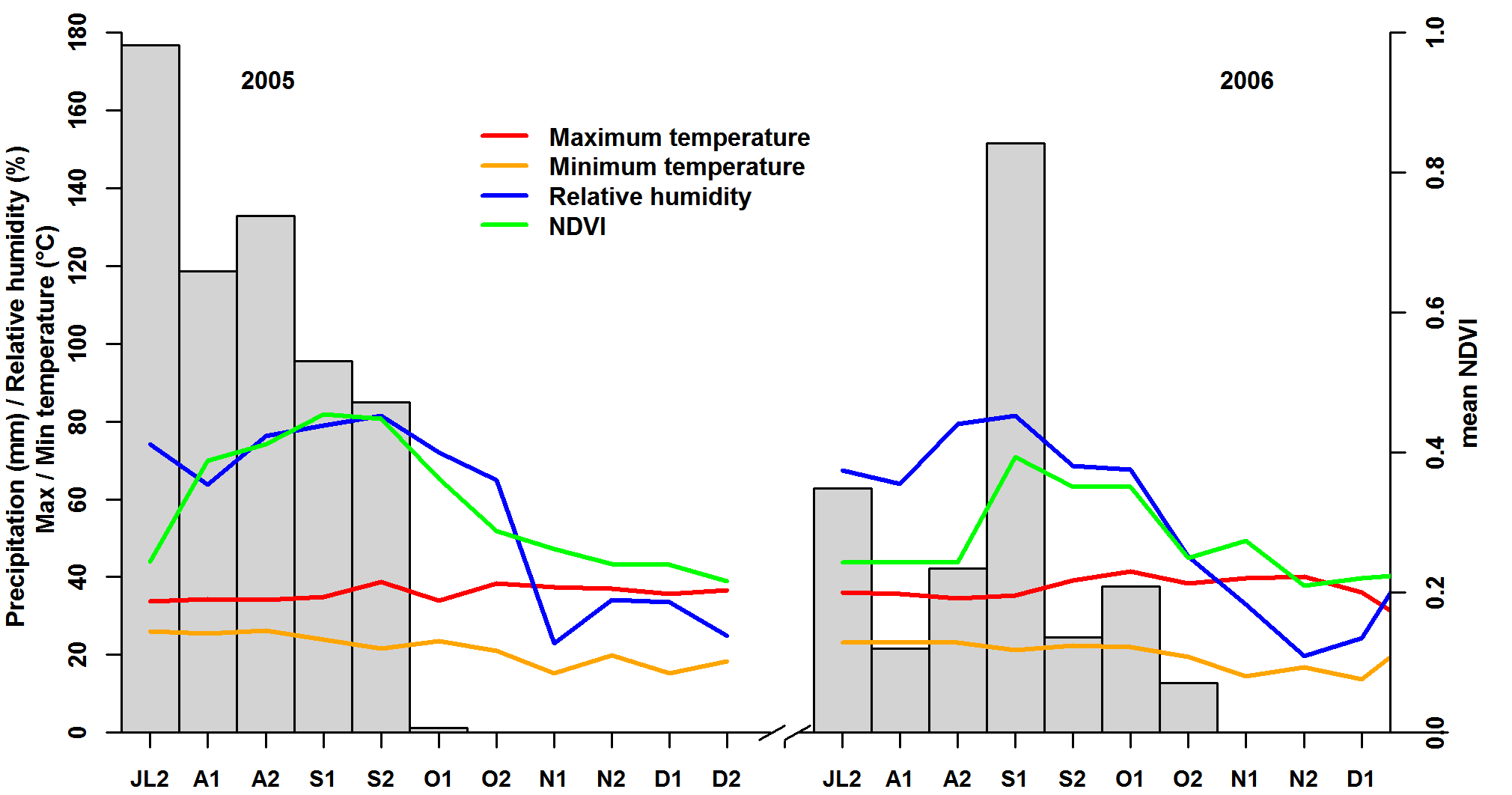

Supplement: Figure S3 — Climate and environmental parameters used in the models. Mean values for NDVI, Maximum/Minimum temperature and relative humidity. Cumulative rainfall 15–20 days prior to trapping. (TIFF) [file pone.0114047.s003.tiff]

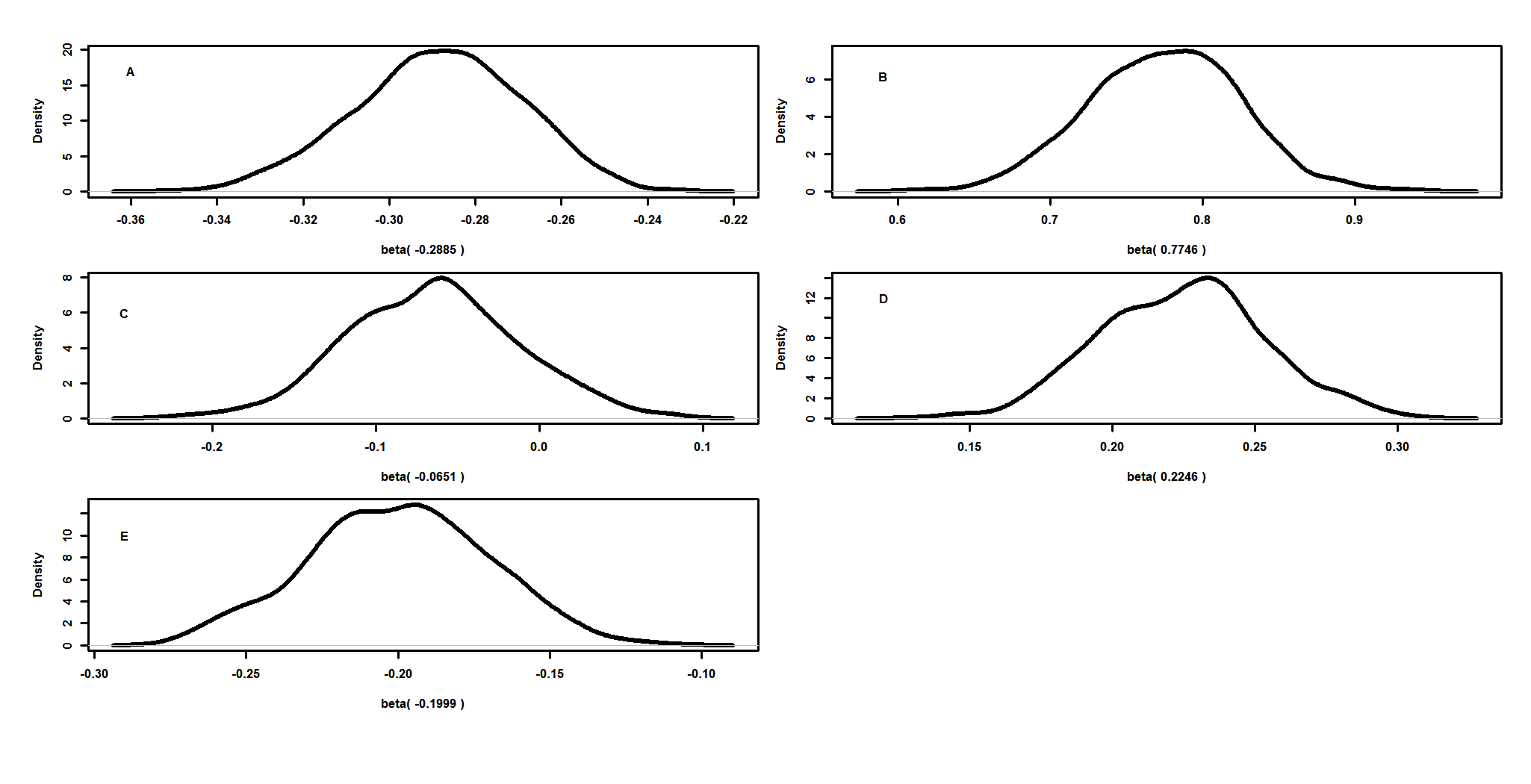

Supplement: Figure S4 — Kernel density estimates for marginal posteriors distributions of parameters associated with variables. Posteriors distributions (posterior mean in parentheses): (A) Maximum temperature, (B) Minimum temperature, (C) Relative humidity, (D) Cumulative rainfall, (E) NDVI for Cx. Poicilipes. (TIFF) [file pone.0114047.s004.tiff]

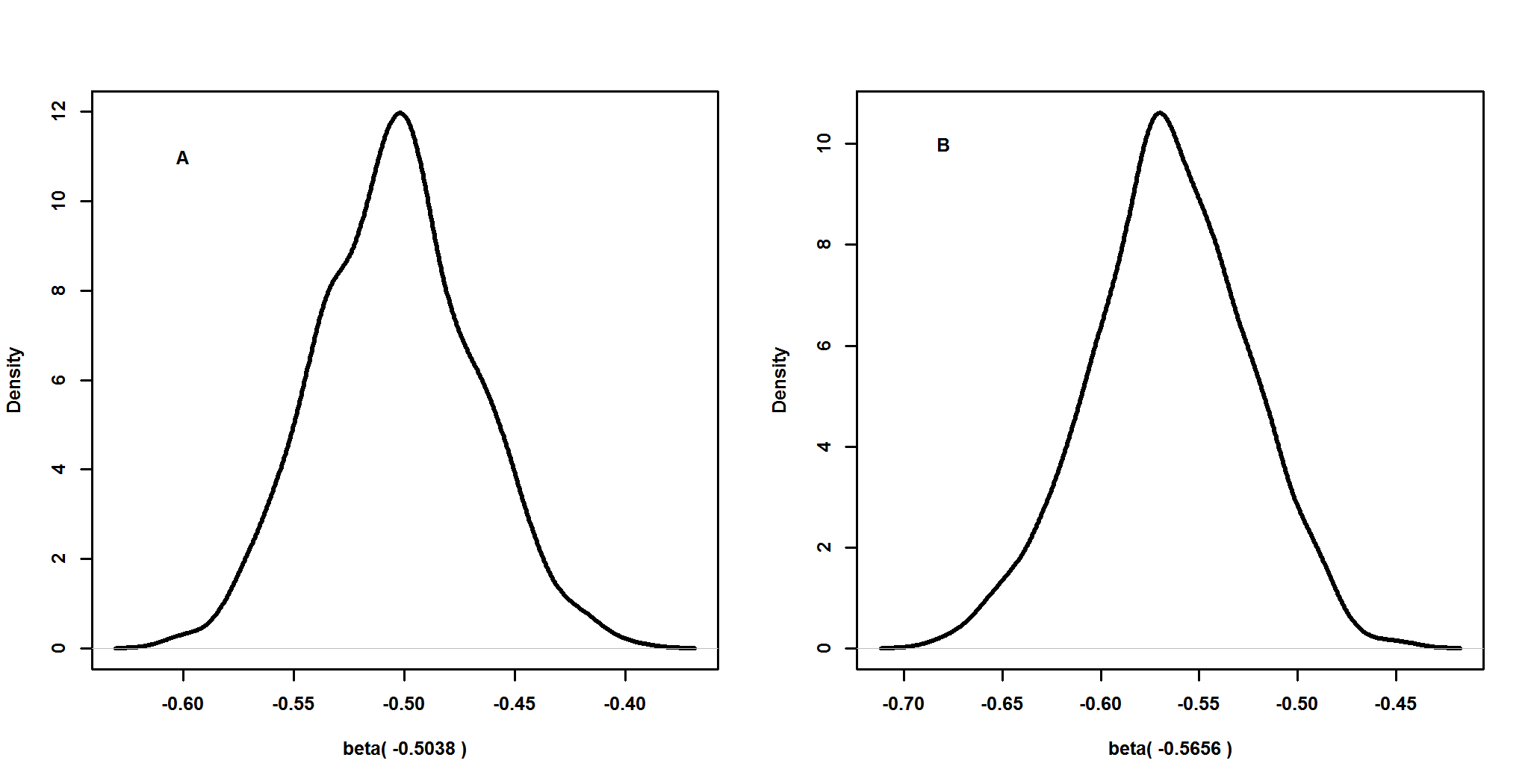

Supplement: Figure S5 — Kernel density estimates for marginal posteriors distributions of parameters associated with variables. Posteriors distributions (posterior mean in parentheses): (A) thermal range (difference between maximum and minimum temperature), (B) Cumulative rainfall for Ae. vexans. (TIFF) [file pone.0114047.s005.tiff]

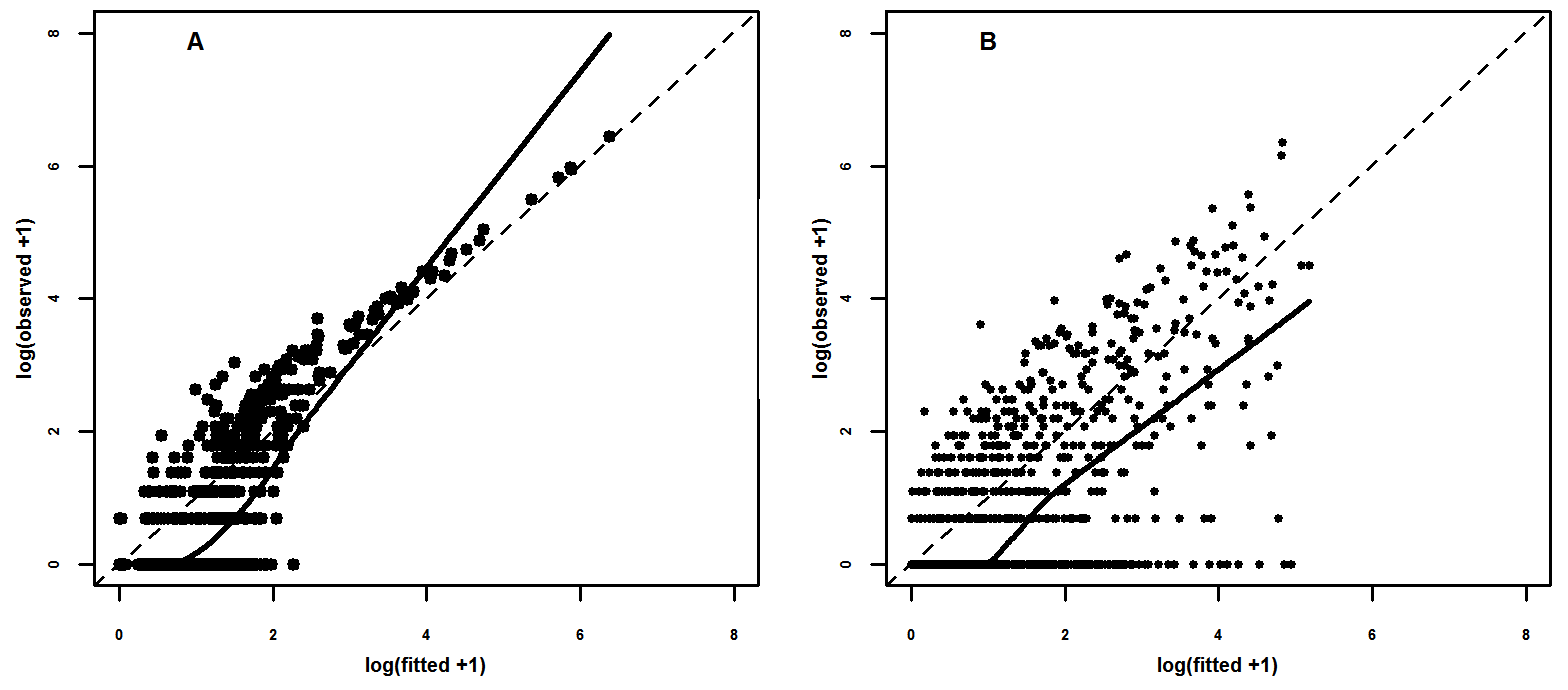

Supplement: Figure S6 — Scatter plot and loess curve. Show observed and predicted data (loess curve in solid line) using GLMM: (A) Cx. poicilipes, (B): Ae. vexans. (TIF) [file pone.0114047.s006.tif]
