# Supplementary material for: Statistical Modeling of the Abundance of Vectors of West African Rift Valley Fever in Barkédji, Senegal
Source: PLoS One. 2014 Dec 1;9(12):e114047. doi: 10.1371/journal.pone.0114047 (PMC4250055; doi:10.1371/journal.pone.0114047)
Supplement: Table S1 — Kendall's rank correlation coefficients and the variance inflation factors (VIFs). Variables correlated at less than 0.8 were maintained in the model. All the VIFs of climatic and environmental variables are well below 10 suggesting that collinearity is no longer a major issue. (DOC) [file pone.0114047.s007.doc]

**Table S1**: **Kendall’s rank correlation coefficients and the variance inflation factors (VIFs) of variables**.

|  | RAIN | TMAX | TMIN | HR | NDVI | VIF |
| --- | --- | --- | --- | --- | --- | --- |
| RAIN | 1 |  |  |  |  | 2.13 |
| TMAX | -0.33 | 1 |  |  |  | 1.34 |
| TMIN | 0.56 | -0.35 | 1 |  |  | 3.78 |
| HR | 0.62 | -0.18 | 0.42 | 1 |  | 5.19 |
| NDVI | 0.46 | 0.0097 | 0.26 | 0.50 | 1 | 2.08 |

RAIN: cumulative rainfall recorded 15-20 days prior to sampling, TMAX: maximum temperature, TMIN: minimum temperature, HR: relative humidity, NDVI: normalized difference vegetation index. All the VIFs are well below 10 suggesting that collinearity is no longer a major issue.
