# Supplementary material for: Statistical Modeling of the Abundance of Vectors of West African Rift Valley Fever in Barkédji, Senegal
Source: PLoS One. 2014 Dec 1;9(12):e114047. doi: 10.1371/journal.pone.0114047 (PMC4250055; doi:10.1371/journal.pone.0114047)
Supplement: Model S1 — WinBUGS codes for the Bayesian hierarchical models. (DOC) [file pone.0114047.s008.doc]

**Model S1**: WinBUGS codes for the Bayesian hierarchical models

**1-Model code for *Aedes vexans***

model{

### Likelihood (N=1738)

for ( k in 1:N ){

Y[k] ~ dpois (lambda[k])

log(lambda[k]) <- beta[1]*RAIN[k] + beta[2]*(TMAX[k] - TMIN[k] )

+ sin((Time[k]/2)*3.14159/4.5)

+ cos((Time[k]/3)*3.14159) + b[Biotope[k]] + q[Time[k]] + eps[k]

}

### Prior for climate and ecological covariates

for (i in 1:2) { beta ~ dnorm (0, 0.1) }

# Prior for land cover classes

for (i in 1:6) { b[i] ~ dnorm(0,10) }

#

for ( i in 1:N) { eps[i] ~ dnorm (0.0, 10)}

# Prior distribution for the autocorrelated fortnight effect

tau ~ dgamma(0.1,0.001)

sigmat <- 1/sqrt(tau)

q[1] <- 0

for (i in 2:11) { q[i] ~ dnorm(q[i-1], tau) }

}

# **2- Model code for *Culex poicilipes***

model{

### Likelihood

for ( k in 1:N ){

Y[k] ~ dpois (lambda[k])

log (lambda[k]) <- beta[1]*TMAX[k] + beta[2]*TMIN[k] + beta[3]*HR[k]

+ beta[4]*RAIN[k] + beta[5]*NDVI[k] + car[Sites[k]]

+ q[Time[k]] + 5*sin((Time[k]-3)*3.14159/6)

}

### CAR prior distribution for spatial random effects:

car[1:79] ~ car.normal(adj[], weights[], num[], precu)

precu ~ dgamma(0.001, 0.001)

sigmau <- 1/precu

### Prior for climate and ecological covariates

for (i in 1:5){ beta[i] ~ dnorm (0.0, 0.1) }

# Prior distribution for the autocorrelated fortnight effect

tau ~ dgamma(0.1,0.001)

sigmat <- 1/sqrt(tau)

q[1]<-0

for (i in 2:11){ q[i] ~ dnorm(q[i-1], tau) }
